# Comparison of Botulinum toxin type A with surgery for the treatment of intermittent exotropia in children

**DOI:** 10.1186/s12886-022-02285-2

**Published:** 2022-02-04

**Authors:** Han Su, Jing Fu, Xiao Wu, Ali Sun, Bowen Zhao, Jie Hong

**Affiliations:** Beijing Tongren Eye Center, Beijing Tongren Hospital, Capital Medical University, Beijing Key Laboratory of Ophthalmology&Visual Science, No.1, Dong Jiao Min Xiang Street, Dongcheng District, Beijing, PR China

**Keywords:** Botulinum toxin type a, Intermittent Exotropia, Strabismus, Childhood

## Abstract

**Background:**

The aim of this study was to observe the effectiveness of botulinum toxin type A (BTA) in the treatment of intermittent exotropia (IXT) in children compared with strabismus surgery.

**Methods:**

One hundred forty-four children with a clear diagnosis of IXT and an indication for surgery were eligible for inclusion. Subjects were divided into two groups based on parental decision: the BTA injection group (injection group) or the conventional surgery group (surgery group). All cases were followed up for 6 months. The primary outcome was a comparison of the success rate (deviation between − 10 and + 10 PD) between the two groups at 6 months after the initial treatment. Secondary outcomes included change in deviation, visual function, and post-surgical complications.

**Results:**

Seventy-two patients were enrolled in each group. At 6-month follow-up, there was no significant difference in the success rate between the injection and surgery groups (52.8% vs 66.7%, *P* = 0.13; postoperative deviation − 12.22 ± 10.80 PD vs − 9.17 ± 10.30 PD, *P* = 0.09). The binocular Visual function, except for near stereoacuity, improved after treatment in both groups, while the fusion recovery rate was higher in the surgical group (68.1% vs 95.8%, *P* < 0.001). Transient complications in the injection group included diplopia, ptosis, and subconjunctival hemorrhage, whereas subconjunctival hemorrhage, conjunctival edema, foreign body sensation, pain, and diplopia were seen in the surgical group. The complications of BTA were relatively mild.

**Conclusions:**

BTA is as effective as surgery in the treatment of IXT in children, but the recovery of the fusion is lower than surgery.

**Trial registration:**

The study has completed the clinical registration on (ChiCTR-INR-17013777).

**Supplementary Information:**

The online version contains supplementary material available at 10.1186/s12886-022-02285-2.

## Background

Intermittent exotropia (IXT) is one of the common eye diseases among children. Surgery has long been considered the treatment of choice for IXT with surgical conditions (horizontal deviation ≥15 PD after complete refractive correction). However, a number of limitations and complications following surgery have gradually come to light, including scar formation and adhesions, difficulties in subsequent secondary surgery, and compromised surgical outcomes due to the low accuracy of deviation measurements in young children. As a result, minimally invasive approaches to IXT have been explored and shown to have a better safety profile.

Botulinum toxin type A (BTA) is a neurotoxin synthesized by *Clostridium botulinum* and was first applied in the treatment of strabismus by Scott [1] in 1973. BTA mediates muscle paralysis by blocking the release of acetylcholine and impeding neuromuscular signaling for approximately 3 months [2] . The rationale for the elimination of strabismus by BTA is to temporarily reduce the tone of the extraocular muscles (EOM) by chemical denervation, thus inducing a specific EOM paralysis. During the restoration of equilibrium of the extraocular muscles, binocular vision is gradually restored and leads to the reconstruction of the alignment position of the eyes, resulting in the treatment of strabismus [3] . Some of the advantages of BTA injection include not affecting the anatomical position of the EOM, maintaining long-term physiological function, reproducibility, simplicity of operation, and few side effects.

The efficacy of BTA injections in the treatment of paralytic strabismus in adults is now widely accepted [4–6] . In recent years, the emerging treatment strategy of treating children with concomitant strabismus by transconjunctival injection of BTA has been proposed. Evidence from small prospective and retrospective clinical trials has shown promising results in acute concomitant esotropia and congenital esotropia. Wan [7] performed BTA injections in 16 children with acute concomitant esotropia, and achieved an 81% success rate 6 months after injection. Campos [8] found that 88% of patients (less than 7 months) who received BTA injections for the first time achieved good alignment. However, the efficacy of BTA in the treatment of IXT is generally inferior to that of esotropia, and there is no consistent results from various studies, with success rates ranging from 38.1 to 86% [9–11] . In addition, the lack of previous comparative studies of BTA injection and surgery in the same clinical study makes the evidence inconclusive [12] The aim of this study was to observe the clinical efficacy of extraocular muscle BTA injection in the treatment of IXT in children and to evaluate the feasibility and safety of this method in comparison with the success rate of surgery.

## Methods

### Study design

This study was a prospective, non-randomized, comparative clinical study. One hundred forty-four children (aged between 3 and 13 years) with IXT attending the strabismus clinic at Beijing Tongren Hospital from April 2018 to October 2019 were eligible for inclusion. Inclusion criteria. (1) IXT patients with indications for surgery (≥ − 15 PD of horizontal deviation after complete correction); (2) age < 18 years; (3) ≤ − 50 PD of horizontal deviation. Exclusion criteria: combination of the following (1) other structural ocular pathologies; (2) vertical strabismus, A-V strabismus, dissociated vertical deviation; (3) nystagmus and amblyopia; (4) neurological or developmental abnormalities; and (5) previous history of strabismus surgery. The study complied with the Declaration of Helsinki. Ethics committee approval was obtained from the Institutional Review Board of Beijing Tongren Hospital, Capital Medical University (TRECKY2018–006). All parents or guardians signed an informed consent form, and we obtained informed consent from all pediatric patients.

### Pre-operative examination

General examination, best corrected visual acuity, and refractive status were performed by a pediatric ophthalmologist. Distance (6 m) and near (33 cm) deviations in primary position were measured using prism and alternate cover test, and deviations that could not be cooperated with were measured by the Krimsky method. Restriction of ductions in horizontal gaze was measured on a 9-point scale (− 4 to + 4), with 0 representing no restriction, − 4 indicating no movement beyond the midline, and + 4 representing extremely strong muscles. Binocular vision at distance fixation, including simultaneous vision, fusion, and distance stereopsis, was examined by synoptophore (CLEMENT-CLARKE, UK, type 2001) (supplementary material shows more details of synoptophore (see Additional file 1)) and near stereopsis at near fixation was performed by Random-dot stereogram.

### Interventions and distribution

Based on parental choice, all patients were divided into two groups: an injection group and a surgical group. Children in the injection group were treated with BTA EOMs injections, while children in the surgery group were treated with conventional surgery.

Injection group. The method of anesthesia was chosen according to the age and cooperation of the child and the opinion of the parents or guardians. General anesthesia was sevoflurane inhalation, and local anesthesia was performed by a drop of proparacaine hydrochloride in the conjunctival sac before injection. BTA was injected into bilateral lateral rectus muscles under electromyography (EMG) guidance without conjunctival incision. BTA (Hengli, Lanzhou Institute of Biological Products; Baotoshi, Aijian Ireland Pharmaceutical Co., Ltd.) was diluted to 100 IU/4 ml with 0.9% saline in a volume of 0.1 ml per muscle. Needle electrodes were inserted into the EOMs under EMG guidance. On emitting a high level acoustic signal, indicating that the neuromuscular junction had been reached, and the injection was performed. Depending on the severity of strabismus, patients received two different doses of treatment. 1.25 IU per muscle was injected in patients with strabismus ≥ − 15 PD and < − 20 PD, while 2.5 IU per muscle was injected in patients with strabismus ≥ − 20 PD and < − 50 PD [13] . For cases with residual strabismus and deviations > − 10 PD during the 6-month follow-up period, repeat injections were performed. All repeat injections were done bilaterally. The dose of repeat injections also followed the method described above.

Surgical group. General anesthesia was performed with sevoflurane inhalation combined with intravenous infusion, and local anesthesia was performed with proparacaine hydrochloride surface anesthesia combined with lidocaine (1%, 0.5 ml) subconjunctival infiltration anesthesia around the surgical muscle. Patients were treated surgically for strabismus according to the preoperative measurement of strabismus deviation. Unilateral lateral rectus recession was performed in children with deviation ≤ − 20 PD, and bilateral lateral rectus recession or unilateral recess-resect was chosen when the deviation was > − 20 PD and ≤ − 50 PD [14] .

### Outcome measurement

Follow-up was set at 1 week, 1 month, 3 months, and 6 months postoperatively (follow-up for children with repeat injections was calculated from the last injection). Ophthalmic examination included measurement of eye position, eye movements, binocular visual function and complications. In indicating the angle of deviation, the symbols (positive and negative) represent only the direction of horizontal strabismus, independent of the preoperative and postoperative period. Therefore, we use the positive value of prism diopters for exotropia pre-operation and the negative values for consecutive esotropia post-operation.

Primary outcome: Success rate at 6 months post-treatment was set as the primary outcome. Treatment success was defined as a final absolute value of horizontal deviation of 10 PD or less (tested by alternate prism cover testing at distance).

Secondary outcomes. (1) angle of deviation (prism diopters, PD) before and after operation; and (2) binocular visual function. The normal range of binocular vision included simultaneous vision between − 3° and + 3°, the presence of fusion and distance stereopsis, and near stereopsis less than or equal to 60 arcsec.

Safety evaluation indicators: complications such as ptosis, diplopia, vertical strabismus, etc.

### Statistical analysis

Statistical analysis was performed using SPSS 23.0 statistical software. Assignment of near stereoacuity: Log was taken for near stereoacuity in arcsecond. “None” was assigned a value of 1600, and 3.20 after taking Log. The prismatic degree and quantitative components of binocular vision before and after treatment were expressed as mean plus standard deviation range and compared by paired t-test. Independent samples t-test was used to compare the results of the injection and surgery groups, including age, angle of deviation, and quantitative components of binocular vision. The Fisher exact test was used to compare the sex ratio, the qualitative component of binocular vision, success rate, and complications between the two groups, described by frequency and percentage. *p* < 0.05 was considered statistically significant.

## Results

### Patient demographics and clinical characteristics

Patient demographics and clinical characteristics are shown in Table 1. A total of 144 subjects were enrolled in this study. The mean age was 7.1 ± 2.5 years (range 3 to 13), and the mean preoperative angle of exodeviation was − 35.9 ± 7.9 PD. Seventy-two patients underwent BTA injection and 72 patients underwent strabismus surgery. No significant differences were found between groups with regard to preoperative clinical factors, including age, gender, angle of deviation, and binocular visual function.

**Table 1:** Preoperative demographic data

Variables	Total (*n* = 144)	Injections (*n* = 72)	Surgery (*n* = 72)	*P*
Age (years)	7.1 ± 2.5	7.0 ± 2.6	7.1 ± 2.4	0.84
Gender (male/female)	73/71	37/35	36/36	1.00
Preoperative angle of exodeviation (PD)	−35.9 ± 7.9	− 35.9 ± 7.3	− 35.8 ± 8.5	0.98
Binocular visual function measured by synoptophore				
simultaneous vision (n, %)	4 (2.8%)	2 (2.8%)	2 (2.8%)	1.00
Fusion (n, %)	91 (63.2%)	45 (62.5%)	46 (63.9%)	1.00
Distance stereopsis (n, %)	50 (34.7%)	26 (36.1%)	24 (33.3%)	0.86
Good near stereopsis (n, %)	57 (39.6%)	34 (47.2%)	23 (31.9%)	0.09
Log arcsec of near stereopsis	2.1 ± 0.4	2.0 ± 0.4	2.1 ± 0.5	0.14

### Treatment success rates in the injection and surgery groups

All patients received 2.5 IU injections based on preoperative strabismus deviation measurements. None received 1.25 IU injections. At 6 months after treatment, there was no significant difference in treatment success (measured as absolute value ≤10 PD) between the injection group (38/72, 52.8%) and the surgical group (48/72, 66.7%, *P* = 0.13).

### Changes in postoperative deviation angle in the surgical and BTA injection groups

**Fig. 1:**
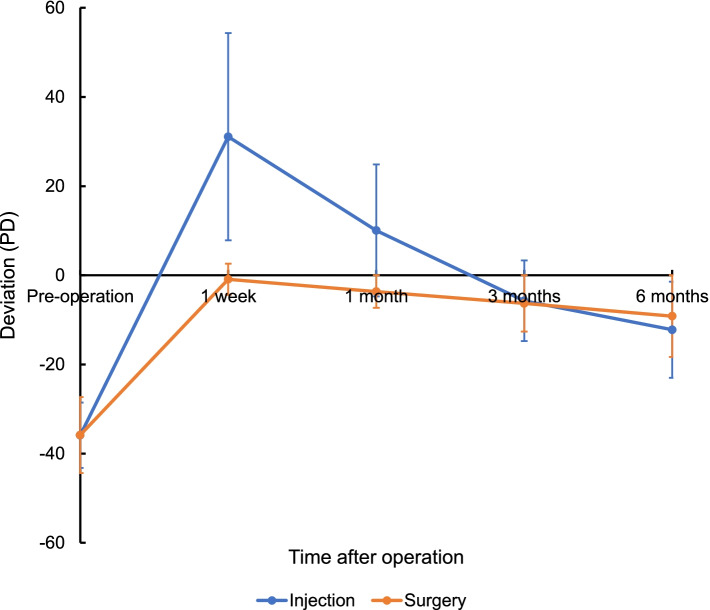
In the injection group, overcorrection was found in children at 1 week after injection. With the extension of follow-up time, alignment gradually recovered and the best effect was shown at 3 months after injection. Six months after injection, recurrence and regression of deviation began to occur. In the operation group, the best effect was shown at 1 week after operation. With progression of time, the angle of deviation gradually increased, which was manifested as regression of IXT

There was a significant difference in the angle of deviation before and after injection (*P* < 0.001) or surgery (*P* < 0.001) at 6 months, and the deviation after treatment was significantly lower than before treatment in both groups. There was no statistically significant difference in the angle of deviation between the two groups at 6 months after treatment (*P* = 0.09) (Table 2). The change in deviation in both groups is shown in Fig. 1. In the injection group, overcorrection was found in children at 1 week after injection. Alignment gradually recovered with longer follow-up, with the best results occurring 3 months after injection. At 6 months after injection, recurrence and regression of deviation began to occur. In the surgical group, the best results appeared 1 week after surgery. The angle of deviation gradually increased with time, showing a regression of IXT.

**Table 2:** Changes in angle of deviation in surgical and BTA injection groups pre- and post- operation (PD)

Time after treatment	Injections	Surgery	*P*
Pre-operation	−35.9 ± 7.3	− 35.8 ± 8.5	0.98
1 week	31.1 ± 23.3	−0.9 ± 3.5	< 0.001
1 month	10.1 ± 14.8	−3.7 ± 6.3	< 0.001
3 months	−5.7 ± 9.1	−6.3 ± 8.2	0.70
6 months	−12.2 ± 10.8	−9.2 ± 10.3	0.09

### Comparison of postoperative binocular vision between the injection and surgery groups

As shown in Table 3, there was no significant difference in the recovery of simultaneous vision (*P =* 0.12) and distance stereopsis (*P =* 0.40) between the two groups at 6 months after treatment, however, the recovery of fusion was significantly higher in the surgical group compared with the injection group (*P* < 0.001). And there was no difference between the two groups in terms of good post-treatment near stereopsis (*P = 0*.09). At 6 months after treatment, the logarithmic arcsecond of near stereopsis was 2.0 ± 0.4 in the injection and 2.0 ± 0.5 in the surgical group. There was no significant difference in the log arcsec of near stereopsis before and after injection (*P* = 0.98)/surgery (*P* = 0.08). And for the log arcsec of near stereopsis after injection and surgery, there was also no significant difference between two groups (*P = 0*.99). After 6 months of treatment, the proportion of patients with good binocular vision increased in both groups compared with pre-treatment (as more visually shown in the supplemental figure material (see Additional file 2)). Although the proportion of patients with good near stereopsis was lower in the BTA group after injection than before, the difference was not statistically significant (*P* = 0.31).

**Table 3:** Comparison of the recovery rate of binocular vision function after treatment between the injection and surgery groups

	Injections	Surgery	*P*
Simultaneous vision (n, %)	23 (33.3%)	34 (47.2%)	0.12
Fusion (n, %)	47 (68.1%)	69 (95.8%)	<0.001
Distance stereopsis (n, %)	31 (44.9%)	38 (52.8%)	0.40
Good near stereopsis (n, %)	27 (39.1%)	39 (54.2%)	0.09
Log arcsec of near stereopsis	2.0 ± 0.4	2.0 ± 0.5	0.99

### Other characteristic changes

All children had normal eye movements before treatment, and all children had an initial EOM force of 0. In the injection group, 47 children had reduced EOM force at the 1-week follow-up (EOM force ranged from − 3 to − 1), and 9 cases had mild limitation of eye movements for 1 month consecutively, with reduced injection muscle force to − 1. After 3 months of injection, all children’s EOM force returned to preoperative baseline. There was no significant oculomotor limitation in the surgery group.

There was a statistically significant difference in the mode of anesthesia between injection and surgery (*P* < 0.001). In the injection group, 33 cases (45.8%) underwent local anesthesia without sedation and 39 subjects (54.2%) under general anesthesia, whereas only 1 case (1.4%) in the surgery group underwent surgery under local anesthesia.

There were no serious complications in the injection group. All children complained of diplopia 1 week after injection, and the diplopia disappeared after 1 month. Temporary ptosis was observed in 19 patients (26.0%), none of whom developed amblyopia. The ptosis disappeared after a median of 1 to 3 months. Subconjunctival hemorrhage was noted in 1 patient, and this symptom disappeared at 1-month follow-up. In the surgery group, some children also complained of diplopia after surgery, usually lasting a few days or weeks. Subconjunctival hemorrhage and conjunctival edema are unavoidable. The edema is usually absorbed within a few weeks, but the hemorrhage is absorbed over a longer period of time and can disappear completely within 3–6 months. In the meantime, due to the surgical wound and sutures, many children develop a foreign body sensation or even pain of the eye, which usually lasts 1–3 months (wound healing).

### Recurrence and relapses in injection group

Fifteen children (20.8% of the injection group) received a second injection (10 under local anesthesia and 5 under general anesthesia), and no further repeat injections were performed. Of the 15 IXT patients, 5 relapsed (absolute value of residual strabismus degree> 10 PD) at 3 months after injection and 10 at 6 months after injection (Table 4). Children who received repeat injections did not complete follow-up after the second injection. Therefore, the results were assessed after the 6-month time point after the first injection, not the second injection. These children were still followed up after the second injection. Our current study only analyzed the results of the primary injection.

**Table 4:** Deviation in subjects with repeated injections

		Deviation (PD).	
Number	Recurrence time/month	Recurrence	Initial
1	6	−30	− 30
2	6	−30	− 28
3	6	−30	−30
4	6	−25	−40
5	3	−35	−48
6	6	−25	−38
7	6	−30	−48
8	6	−23	−28
9	3	−30	−40
10	6	−25	−48
11	6	−25	−40
12	6	−28	−35
13	3	−15	−28
14	3	−23	−35
15	3	−25	−48

Twenty other patients in the injection group relapsed but did not undergo a second injection. Of these subjects, 8 patients relapsed at 3 months after injection and 12 patients relapsed at 6 months after injection. The mean deviation of relapse was −18.2 ± 6.3 PD (range − 12 to − 35 PD). Considering their relatively low relapse deviation, the physicians agreed to observe temporarily after communication with the parents and then decide whether to repeat the injection according to the changes.

## Discussion

Intermittent exotropia is the most common type of strabismus in East Asian children [15–17]. One of its greatest dangers is the progressive loss of binocular visual function, which may not be recovered without timely intervention. For decades, surgery has been considered the standard procedure for the treatment of intermittent exotropia in children. Several limitations have emerged in the implementation of the procedure, including postoperative scar adhesions, variable reliability and accuracy, and inconvenient general anesthesia. In addition, the high recurrence rate of IXT (22–59%) is a major problem [9, 10]. Therefore, a second or even multiple surgeries may be required, which increases the suffering of the child. Recently, the use of BTA in ophthalmic surgery has brought light to a new approach for the treatment of IXT and has been used as an initial, secondary and adjunctive treatment in several studies [18].BTA injection can control eye position and maintain some binocular visual function through chemical denervation, thus delaying the age of surgery and reducing the number of operations. Previously, most of the research were based on small sample sizes and single-group observational studies (usually around 30 subjects), such as Spencer [11], Wu [13], and Etezad [14] who reported stable success in 69, 76.67, and 86% of patients, respectively. However, the lack of comparison between BTA and surgery in case-control conditions leads to controversial conclusions.

In the present study, we enrolled 144 children with IXT with indications for surgery and divided them into a BTA injection group and a surgery group. The treatment success rate at the last follow-up at 6 months in the injection group, evaluated with eye position, was 52.8%, which was as effective as the surgery group (66.7%, *P* = 0.13), but lower than previous studies. A variety of reasons may be involved, including different evaluation metrics for surgical success, the preoperative angle of deviation in pediatric IXT patients, and the inclusion of repeat injections. Previous studies [19] showed no significant dependence of the efficacy of BTA injection on the dose of injection, and the dose in the above-mentioned study was 2.5 IU (the same as in the present study), so the dose of BTA was not considered to be a factor affecting the success rate.

Overall, there is a generally accepted criterion for success in intermittent exotropia surgery. As mentioned in the review, several articles used “within +4 PD esotropia to -10 exophoria” [12, 20, 21] However, previous BTA literature on childhood strabismus mostly used the success criterion of “+/− 10 PD”. In our study, success was set as a deviation between − 10 PD and + 10 PD [22], which is consistent with Scott’s original evaluation in the study of BTA for strabismus in children [19]. It is also consistent with other studies reporting evaluation metrics regarding BTA injections for the treatment of concomitant strabismus in children, in which the deviation angle in the success criteria for strabismus treatment ranged from 8 PD to 12 PD, with 10 PD being the most [7, 8, 11, 13, 14]. In addition, previous studies have shown that large deviation degree can affect treatment outcome. Lennerstrand G stated that patients with deviation degree>40 PD had poor BTA treatment and required repeated injections [18]. Spencer [11] and Etezad [14] reported that the mean preoperative deviation of patients was − 29.1 ± 1.7 PD and 30.9 ± 7.1 PD, respectively, with success rates of 69 and 86%. In contrast, in the present study, the preoperative deviation was − 35.9 ± 7.3 PD, which is greater than the above-mentioned data in the literature. In addition, 40.3% (29 subjects) had a deviation between 40 PD and 50 PD, which may affect the success rate. A third reason is that when assessing success rates, previous literature tends to include final follow-up results for repeat injections. The study by Spencer et al. [11] reported 12 patients who received repeat injections (seven patients received two BTA injections and five patients received three injections), nine of whom continued to show favorable eye alignment after more than 1 year of follow-up. We include in this article only the results of the initial single injection. The final follow-up of children with repeated injections has not been completed, and the final results are not known because many children did not complete the 6-month follow-up after the second injection, which may also be a factor in the lower orthostatic rate than previously reported.

Binocular vision is an important indicator of visual performance and one of the key mechanisms for eye position control in patients with IXT. Several articles have highlighted the relationship between satisfactory treatment outcome and recovery of binocular vision. McNeer et al. showed that early injection of BTA in binocular medial rectus for patients with congenital esotropia reconstructed motor and sensory fusion function with long-term effects comparable to surgical correction [23]. Etezad et al. reported that 66.7% of patients with IXT achieved excellent fusion function at 6-month follow-up after BTA injection [14]. In our study, 68.1% of patients in the injection group achieved good fusion control at the 6-month follow-up after treatment, which was lower than that of the surgery group. The reason for this may be due to the greater overcorrection (consecutive esotropia) early in the child after BTA injection, which may impair fusion. If consecutive esotropia persists, patients may experience severe loss of sensory function; diplopia, suppression, amblyopia, and loss of binocular vision may occur [24, 25]. In our study, overcorrection (consecutive esotropia) lasted for a maximum of 3 months, with most patients lasting 1 month. Short-term overcorrection did not cause other damage to the patients, but the establishment of fusion was worse than in surgical patients. For simultaneous vision and distance stereopsis 6 months after treatment, there were no statistical differences between the injection and the surgery groups. For stereoacuity measurements, both the injection and surgery groups showed a considerable preference for near stereopsis before and after treatment, which is consistent with the clinical features of IXT. At 6 months post-treatment, binocular visual function improved at all levels compared to baseline, except for near stereopsis.

Complications of BTA injections for strabismus have been reported to include ptosis, amblyopia, overcorrection, and vertical strabismus [26] In our study, diplopia, ptosis, and subconjunctival hemorrhage were observed in 72 subjects, 19 subjects and 1 subject, respectively. These complications gradually diminished from 1 month to 6 months during the follow-up period. The upper eyelid ptosis did not cover the pupil, and these symptoms disappeared within 1 to 3 months. Therefore, no children with occlusion amblyopia have been observed. Compared to the complications of surgery, the complications of BTA injections are milder, shorter in duration, and less painful for the child.

Pediatric general anesthesia poses significant considerations for parents and adds to the burden of the procedure. Compared to traditional extraocular muscle surgery, approximately half of the children in the BTA injection group could be performed with local anesthesia and a second injection could be performed. This also demonstrates that BTA injections are significantly more convenient than surgery, while reducing the demand on medical resources and improving the operability of repeat injections. Furthermore, in China, parents prefer local anesthesia for surgery. BTA not only facilitates local anesthesia injection, but also increases the possibility of local anesthesia surgery, as the binocular visual function can be preserved after BTA injection and the age of surgery can be postponed, which is more in line with the needs of Chinese parents.

### Limitations

This study has several limitations. First, and most importantly, the study was limited by short-term follow-up. Children with well-controlled fusion with intermittent exotropia and no amblyopia are usually examined every 6 to 12 months [25]. The follow-up period for BTA injections reported in the literature ranges from 6 to 44 months [7, 8, 11, 13, 14], with a minimum of 6 months of follow-up according to Scott’s recommendations [19]. Based on this, a 6-month follow-up was chosen as the final time point for observing the results in this study. However, the short follow-up period does represent a major limitation, and continuous follow-up is still needed to study the long-term efficacy of BTX injection in intermittent exotropia. In addition, the timing and dose of repeat injections and the relationship between baseline deviation and repeat injections are worth exploring. Tengtrisorn [27] used twice the initial dose of BTA injection as a second dose for the treatment of esotropia in children, achieving success in 72.7% of cases. And the sample size is still small, and the researchers will continue to expand the sample size for further studies.

## Conclusions

In conclusion, BTA injection is as effective as surgical treatment in children with low to moderate deviation (− 20 PD to − 50 PD) compared to traditional surgery. At the same time, the children’s binocular visual function can be maintained, although the recovery of fusion is not as good as surgery. BTA injection, as a novel alternative method for the treatment of IXT in children, still has a promising application because of its simplicity, reproducibility, minimally invasive, low requirements for anesthesia care, and reliable safety.

## Supplementary Information

**Additional file 1.**

**Additional file 2.**

## Data Availability

The datasets used and/or analysed during the current study are available from the corresponding author on reasonable request.
