# Supplementary material for: Comparison of Botulinum toxin type A with surgery for the treatment of intermittent exotropia in children
Source: BMC Ophthalmol. 2022 Feb 4;22:53. doi: 10.1186/s12886-022-02285-2 (PMC8815233; doi:10.1186/s12886-022-02285-2)
Supplement: Supplementary file 1 — Additional file 1. [file 12886_2022_2285_MOESM1_ESM.doc]

**Comparison of** **Botulinum Toxin Type A with Surgery for the Treatment of Intermittent Exotropia in Children**

Han Su^1^, Jing Fu^1^, Xiao Wu^1^, Ali Sun^1^, Bowen Zhao^1^, Jie Hong^1^

1. Beijing Tongren Eye Center, Beijing Tongren Hospital, Capital Medical University, Beijing Key Laboratory of Ophthalmology＆Visual Science, Beijing, China

Corresponding author:

Dr. Jing Fu, M.D., PhD, MPH

Ophthalmology Department of Beijing Tongren Hospital, Capital Medical University
Add: No.1, Dong Jiao Min Xiang Street, Dongcheng District, Beijing, P.R.China

Email: [fu_jing@126.com](mailto:fu_jing@126.com)

The file shows figures for the measurement of synoptophore. Synoptophore (Fig. a) is an instrument used to test the binocular vision function, eye movement mobility, and quantify the deviations of strabismus [1,2,3]. In our study, synoptophore was mainly used to evaluate the patient's binocular vision, that is, simultaneous vision (Fig. b), fusion (Fig. c) and distance stereopsis (Fig. d). After inserting different pictures in the instrument, the examiner can adjust the instrument so that the examinee can see different object images with both eyes at the same time or separately, so that the simultaneous vision and fusion range can be examined. And through a set of three-dimensional pictures, the examiner can directly check whether the patient has stereoscopic vision. [4,5] Synoptophore is widely used in Asia as an qualitative and quantitative testing equipment for binocular vision.


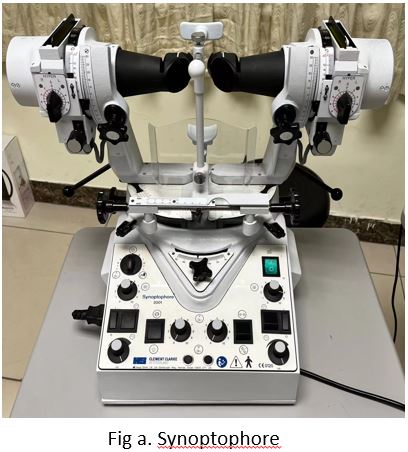


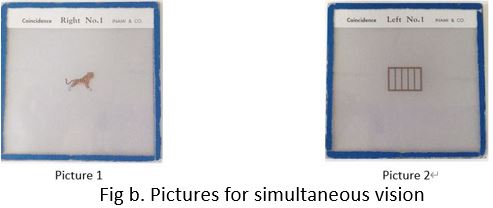


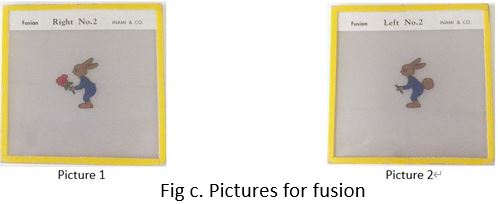


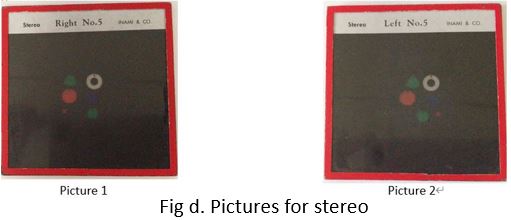


References

1. Georgievski Z. Synoptophore versus prism and cover test measurements in strabismus.: A question of instrument convergence? Strabismus. 1995;3(2):71-7.

2. Tidbury LP, O’Connor AR, Wuerger SM. The effect of induced fusional demand on static and dynamic stereoacuity thresholds: the digital Synoptophore. BMC Ophthalmol. 2019 Jan 7;19(1):6.

3. Xue Z, Min X, Wang J, Zhu Y, He S, Liu K, Ding Y. Clinical Manifestations, Diagnosis, and Surgery of Inferior Oblique Muscle Ectopia. J Ophthalmol. 2020:3039180.

4. Lewis MM. An investigation of "normal" on the synoptophore. Br J Ophthalmol. 1946;30(12):749-57.

5. Foster J, Jackson AS. Aphakic stereoscopic vision, with a note on the synoptophore. Br J Ophthalmol. 1933;17(2):98-102.
