# Supplementary material for: Comparison of Botulinum toxin type A with surgery for the treatment of intermittent exotropia in children
Source: BMC Ophthalmol. 2022 Feb 4;22:53. doi: 10.1186/s12886-022-02285-2 (PMC8815233; doi:10.1186/s12886-022-02285-2)
Supplement: Supplementary file 2 — Additional file 2. [file 12886_2022_2285_MOESM2_ESM.pptx]

## Slide 1
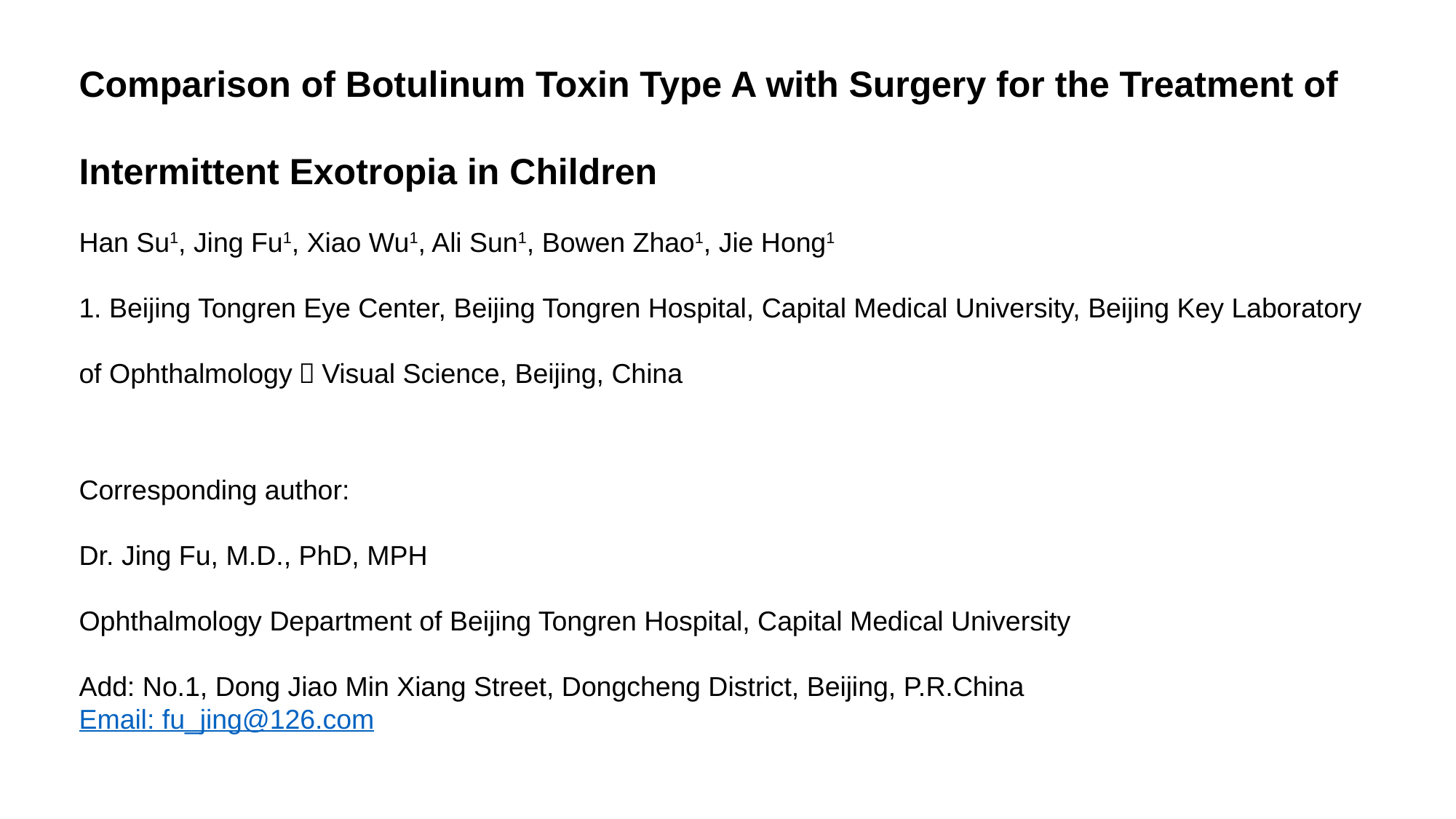

Comparison of Botulinum Toxin Type A with Surgery for the Treatment of Intermittent Exotropia in Children
Han Su1, Jing Fu1, Xiao Wu1, Ali Sun1, Bowen Zhao1, Jie Hong1
1. Beijing Tongren Eye Center, Beijing Tongren Hospital, Capital Medical University, Beijing Key Laboratory of Ophthalmology＆Visual Science, Beijing, China
Corresponding author:
Dr. Jing Fu, M.D., PhD, MPH
Ophthalmology Department of Beijing Tongren Hospital, Capital Medical University
Add: No.1, Dong Jiao Min Xiang Street, Dongcheng District, Beijing, P.R.China
Email: fu_jing@126.com

## Slide 2
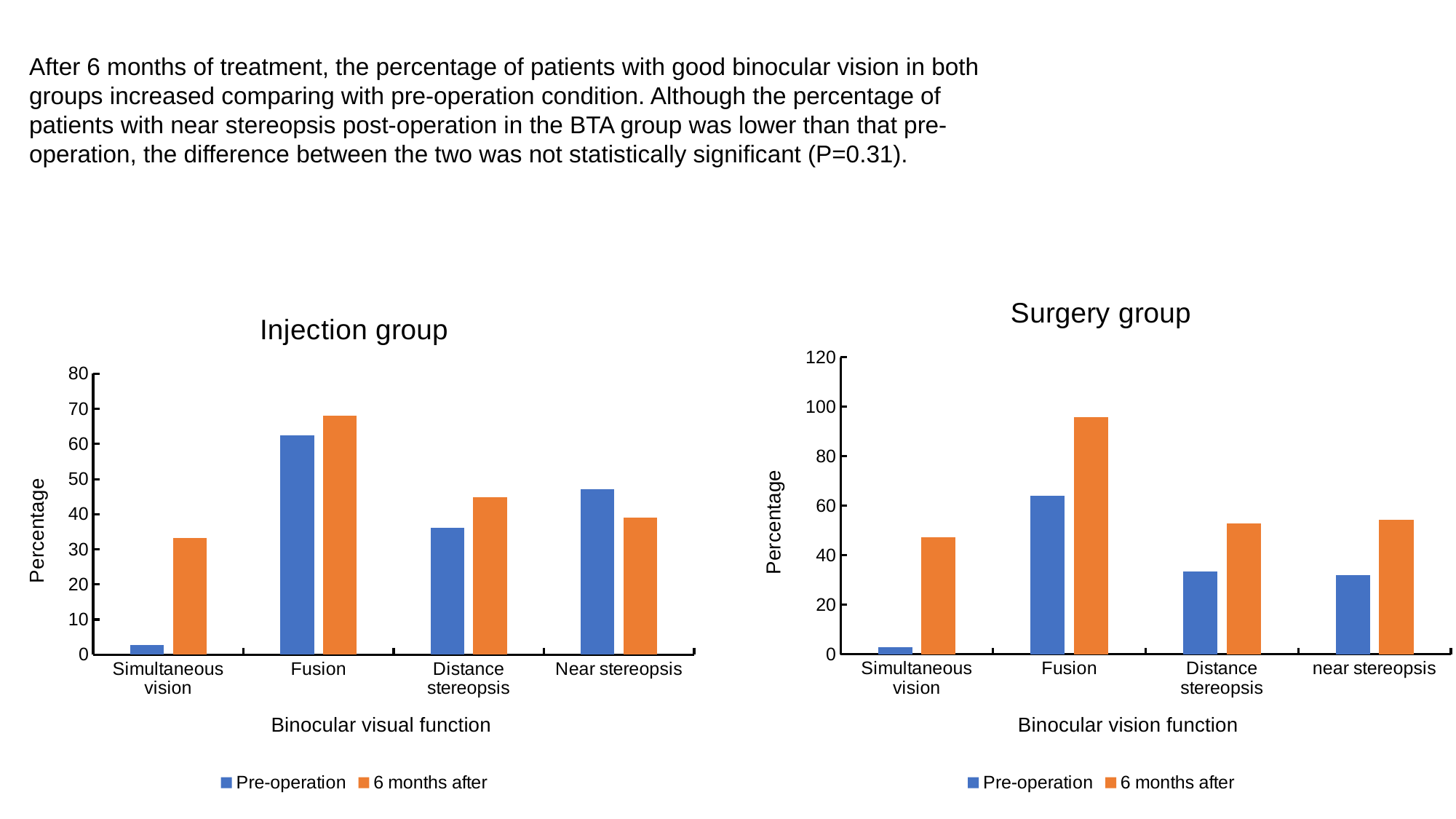

After 6 months of treatment, the percentage of patients with good binocular vision in both groups increased comparing with pre-operation condition. Although the percentage of patients with near stereopsis post-operation in the BTA group was lower than that pre-operation, the difference between the two was not statistically significant (P=0.31).
### Chart: Surgery group
| Category | Pre-operation | 6 months after |
|---|---|---|
| Simultaneous vision | 2.8 | 47.2 |
| Fusion | 63.9 | 95.8 |
| Distance stereopsis | 33.3 | 52.8 |
| near stereopsis | 31.9 | 54.2 |
### Chart: Injection group
| Category | Pre-operation | 6 months after |
|---|---|---|
| Simultaneous vision | 2.8 | 33.3 |
| Fusion | 62.5 | 68.1 |
| Distance stereopsis | 36.1 | 44.9 |
| Near stereopsis | 47.2 | 39.1 |
